# Doubt in the Insula: Risk Processing in Obsessive-Compulsive Disorder

**DOI:** 10.3389/fnhum.2016.00283

**Published:** 2016-06-14

**Authors:** Judy Luigjes, Martijn Figee, Philippe N. Tobler, Wim van den Brink, Bart de Kwaasteniet, Guido van Wingen, Damiaan Denys

**Affiliations:** ^1^Department of Psychiatry, Academic Medical Center, University of AmsterdamAmsterdam, Netherlands; ^2^Brain Imaging Center, Academic Medical Center, University of AmsterdamAmsterdam, Netherlands; ^3^Laboratory for Social and Neural Systems Research, Department of Economics, University of ZurichZurich, Switzerland; ^4^Netherlands Institute for Neuroscience, The Royal Netherlands Academy of Arts and SciencesAmsterdam, Netherlands

**Keywords:** insula, obsessive-compulsive disorder, risk processing, risk avoidance, fMRI

## Abstract

Extensive cleaning or checking of patients with obsessive-compulsive disorder (OCD) are often interpreted as strategies to avoid harm and as an expression of the widespread belief that OCD patients are more risk-averse. However, despite its clinical significance, the neural basis of risk attitude in OCD is unknown. Here, we investigated neural activity during risk processing using functional magnetic resonance imaging and simultaneously assessed risk attitude using a separate behavioral paradigm in OCD patients with different symptoms versus healthy controls (HCs). We found opposite insula responses to high versus low risk in OCD patients compared to HCs: a positive correlation between insula activity and risk-aversion in patients versus a negative correlation in controls. Although OCD patients overall were not more risk-averse than controls, there were differences between subgroups of OCD patients: patients with doubt/checking symptoms were more risk-averse than other patients. Taken together, OCD patients show a reversed pattern of risk processing by the insula compared to HCs. Moreover, the data suggest that increased activation of the insula signals an abnormal urge to avoid risks in the subpopulation of OCD patients with doubt and checking symptoms. These results indicate a role for the insula in excessive risk-avoidance relevant to OCD.

## Introduction

Risk and the need to assess risk pervade our daily life. The outcome of choices is not always certain but different outcomes can occur probabilistically. The variance in value of these possible outcomes is a straightforward measure of the risk involved in the decision. For instance, if the choice can lead to either a very positive or a very negative outcome for the individual, there is a higher variance and therefore a higher risk than if the choice can lead to a moderately positive or a moderately negative outcome. Moreover, how the level of risk influences one’s choice differs between individuals. Some may prefer low risk over high risk, for example because they overweigh the relative loss provided by the worst outcome, whereas others may be drawn to choices that involve high risk. Overall, people tend to prefer safer options, at least when the stakes are high (Pratt, 1964). This tendency toward risk avoiding strategies may have evolved because they may have promoted survival in cases where negative outcomes were life-threatening (Hintze et al., 2015). However, too much risk aversion may lead to suboptimal behavior and may be related to psychopathology.

Clinical observations and stereotypical portraits of obsessive-compulsive disorder (OCD) in the media have led to the common-sense belief that these patients have an abnormal risk assessment: they perceive more risk, are more averse to risk, and therefore develop compulsions to prevent or avoid these perceived dangers such as contamination or harm to self or others. This believe has resonated with scientists (Steketee and Frost, 1994) and is in agreement with the finding that similar brain regions are involved in risk processing and OCD: striatum, insula, prefrontal cortex, and cingulate cortex [e.g. OCD: (Figee et al., 2011; Jung et al., 2011; Stern et al., 2011; Cocchi et al., 2012; Remijnse et al., 2013), risk processing (Preuschoff et al., 2006, 2008; d’Acremont and Bossaerts, 2008; Christopoulos et al., 2009; Tobler et al., 2009; Mohr et al., 2010; Holper et al., 2014)]. However, very little is known about the role of risk attitude and its neural correlates in OCD and available studies are inconsistent: OCD patients were either more averse to risk and showed increased amygdala activation after having made a risky choice (Admon et al., 2012) or they showed no difference in the proportion of risky choices compared to healthy controls (HCs) (Starcke et al., 2010) or difference in risk avoidance did not explain any difference in choice behavior between OCD patients and HCs (Gillan et al., 2013). Additionally, OCD is a heterogeneous disorder and it has been suggested that abnormal (i.e., heightened) risk perception may be more associated with a specific subtype of OCD characterized by worry about harm and checking compulsions (Rasmussen and Eisen, 2002). However, the relation between risk attitude in OCD and the underlying neural mechanisms of risk processing has never been investigated.

In the present study we measured risk attitude and brain activation during risk processing separately using two behavioral paradigms that exposed participants to more or less risk and functional magnetic resonance imaging (fMRI). This design enabled us to compare behavioral and neural differences in risk processing between groups and moreover to investigate whether risk attitude affected neural processing of risk differently in OCD patients compared to HCs. We hypothesize that OCD patients will be more aversive toward risk and show abnormalities in risk related brain regions during risk processing. In particular, based on the central role of the insula and the lateral prefrontal cortex in processing risk and risk attitude (Preuschoff et al., 2006; Burke and Tobler, 2011; Holper et al., 2014), we expect to find differences in risk-processing between patients and HCs in these regions.

## Materials and Methods

### Participants

A total of 18 OCD patients were recruited at the Psychiatric Department of the Academic Medical Center of the University of Amsterdam and 16 control subjects were recruited from the community. Due to a hardware problem the data from the behavioral paradigm of three controls and one patient were lost. Therefore, the behavioral data and regression analysis for risk attitude and fMRI contrasts were based on 17 OCD patients and 13 control subjects. The groups were matched for age, pre-morbid intellectual functioning (IF) and gender (see **Table 1**). The diagnosis of OCD was established by a psychiatrist and confirmed by the Mini International Neuropsychiatric Interview (Sheehan et al., 1998; van Vliet and de Beurs, 2007) according to DSM-IV criteria. Patients with a history of psychosis, bipolar disorder, developmental disorder, traumatic brain injury, or substance dependence were excluded from the study. The control group consisted of medication-free, healthy subjects without a history of OCD or any other psychiatric disorder. The study was approved by the Medical Ethics Committee of the Academic Medical Center of the University of Amsterdam and all participants gave written informed consent.

**Table 1:** Demographic and clinical data of patients and healthy controls (HCs).

Total group	Patients (*N* = 18)		Controls (*N* = 16)		Difference
	Mean	Range	Mean	Range	*P*-value
Age (y)	34 (6.8)	23–54	36 (9.4)	22–58	0.599
Gender (M:F)	6:12		4:12		0.595
Pre-morbid IF	107 (5.4)	98–118	109 (4.9)	100–116	0.212
HAM-A	11.17	0–26	0.44	0–2	0.000
HAM-D	9.06	0–24	0.69	0–3	0.000
YBOCS	23.89	12–33	0	0	0.000

**Analyzed group**	**Patients (*N* = 17)**		**Controls (*N* = 13)**		**Difference**
	**Mean**	**Range**	**Mean**	**Range**	***P*-value**

Age (y)	34 (7.0)	23–54	34 (8.4)	22–48	0.978
Gender (M:F)	6:11		2:11		0.222
Pre-morbid IF	107 (5.1)	100–118	108 (4.6)	100–114	0.777
HAM-A	11.82	0–26	0.38	0–2	0.000
HAM-D	9.59	2–24	0.77	0–3	0.000
YBOCS	23.47	12–33	0	0	0.000

*HAM-A, Hamilton Ratings Scale for Anxiety; HAM-D, Hamilton Ratings Scale for Depression; YBOCS, Yale–Brown Obsessive-Compulsive Scale.*

### Study Procedure

On the day of testing subjects were first assessed for clinical and demographic data, then they conducted a computer task outside the scanner to assess risk attitude and finally they carried out a separate risk processing paradigm during an fMRI scanning session.

### Assessments

#### Clinical Characteristics

Obsessive-compulsive disorder symptoms and OCD severity were assessed using the Yale–Brown Obsessive-Compulsive Scale and the related symptom checklist (Y-BOCS, Y-BOCS-SC (Goodman et al., 1989). The presence of anxiety and depression symptoms was assessed with the Hamilton Rating Scales for Anxiety [HAM-A (Hamilton, 1959)] and Depression [HAM-D (Hamilton, 1960)]. Pre-morbid IF was estimated using the Dutch version of the National Adult Reading Test [DART (Schmand et al., 1991)]. As expected, patients showed significantly more depression, anxiety, and obsessive-compulsive symptoms than controls (**Table 1**).

Ten patients were treated with serotonin reuptake inhibitors, one with a tricyclic antidepressant (clomipramine) and seven patients were unmedicated.

#### Measuring Risk Attitude (Outside Scanner)

Individuals differ in their choice behavior in accordance to their risk attitude: with similar expected value, risk-averse individuals prefer a low risk gamble over a high risk gamble. Risk can be mathematically defined as the variance of possible outcomes following the mean-variance approach of finance theory (Markowitz, 1959). According to this approach, we operationalized a low risk gamble by a smaller variance between two outcomes of equal probability compared to a high risk gamble. A risk-averse individual my prefer a low risk gamble, for example, because they overweigh the relative loss provided by the worst outcome whereas risk-seeking individuals prefer gambles with higher risk and may overweigh the relative gain provided by the best outcome (Christopoulos et al., 2009).

To determine risk attitude, each participant performed a computer task before the scanning session previously used by (Christopoulos et al., 2009). In each trial participants were presented with a gamble (two amounts with equal probability) and a safe alternative (one amount) that they had to choose from. There were three blocks with three different gambles of different risk level: 40 and 60 (low risk), 30 and 70 (medium risk), 10 and 90 (high risk), all with the same expected value of 50 and the same probability of 0.5. In each block the gamble was kept constant while the safe amount varied in each trial according to a staircase method (parameter estimation by sequential testing: PEST) to establish the safe amount for which participants were indifferent between the gamble and safe alternative. The safe amount for which participants were indifferent between the gamble and safe amount is called the certainty equivalent (CE). For each of the three gambles (low, medium, and high risk) the CE was established. When the risk increases between the gambles a risk-sensitive person adapts their CE. Risk-aversion is the difference between the low risk CE minus the high risk CE and reflects how much the individual is influenced by risk. A person with no difference between the CE of a high and low risk gamble is unaffected by the level of risk and is therefore called risk-neutral. By contrast, a person is called risk seeking when the CE of the high risk gamble is larger than the CE of the low risk gamble and risk-averse when the inverse is true. For example, for the low risk gamble (40 and 60), a risk-averse person may have a CE of 45 and for the high risk gamble (10 and 90) a CE of 35, resulting in a risk-aversion of 45–35 = 10 (Christopoulos et al., 2009).

#### Risk Processing Paradigm (Inside Scanner)

**FIGURE 1:**
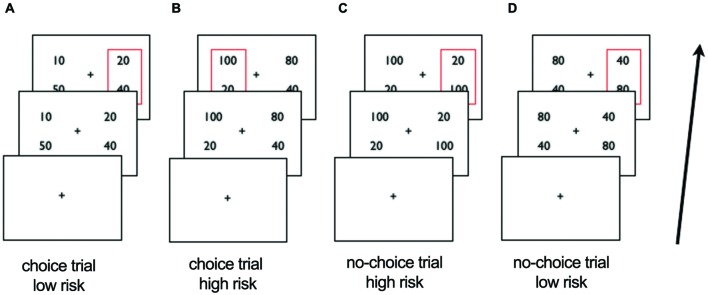
**Schematic overview of scanner task.** After viewing a fixation cross participants are presented with two gambles in the first period and choose the left or right side. In choice trials **(A,B)** the gambles differ in risk, in no-choice trials **(C,D)** they do not. After 4.5 s the choice of participants is represented by a red square around the chosen gamble in slide 2. Examples of trials: **(A)** choice trial, participant chooses low risk gamble **(B)** choice trial, participant chooses high risk gamble; **(C)** no-choice trial, participant ‘chooses’ high risk gamble **(D)** no-choice trial, participant ‘chooses’ low risk gamble.

We adapted a paradigm used previously (Christopoulos et al., 2009; Tobler et al., 2009) which probes monetary risk processing in both choice and non-choice situations. First, participants were presented for 4.5 s with two gambles, both of which were made up of two monetary amounts and matched for expected value. One gamble was presented on one side and the other on the other side of the central fixation cross and participants were instructed to choose one of the two sides by button press within the 4.5 s (**Figure 1**). The response time (RT) (i.e., the time from the onset of the gambles until the button press) was measured in each trial. When participants did not respond in time they were presented with a red cross indicating their late response and the trial was repeated. Second, when participants responded in time, the gambles from the first period were presented with a red rectangular around the gamble of the chosen side for 1 s. During the intertrial interval, which varied between 2.7 and 7.4 s, a fixation cross was shown.

In each trial, participants had a 50% chance to win either amount on the chosen side (i.e., one of the two components of the chosen gamble). When, for example, 50 and 10 was shown on the left side and 40 and 20 on the right side, choosing the left side lead to a red rectangular around 50 and 10 on the left side and a 50% chance of winning 50 and a 50% chance of winning 10. To control for the possibility of outcome related activation contaminating risk-related activation and impacting subsequent choice behavior we did not show the outcomes of each choice. The participants were informed that at the end of the experiment, one trial would be chosen randomly and played out to determine their payoff in Euros.

In each choice trial, the presented monetary amounts made up a high or a low risk gamble, such that one side was riskier than the other. The high risk gamble was defined as 66% gain or loss relative to the expected value, whereas the low risk gamble consisted of a 33% gain or loss relative to the expected value. The gambles on each side had an expected value of either 30 (i.e., the low risk gamble had the possible outcomes of 20 and 40 whereas the high risk gamble has the possible outcomes of 10 and 50) or 60 (i.e., possible outcomes of low risk gamble: 40 and 80; possible outcomes of high risk gamble: 20 and 100).

As in the original task adapted from Christopoulos et al. (2009) we included no-choice situations to expose all participants to both high and low risk. In no-choice situations the gambles on both sides were exactly the same such that participants were forced to undergo the presented risk by selecting one of the two sides (i.e., on each side 10 and 50 or 20 and 100 for high risk and 20 and 40 or 40 and 80 for low risk). In contrast, in choice situations participants could avoid exposure to specific risk levels, resulting in only high risk gamble choices (for risk seeking participants) or only low risk gamble choices (for risk-averse participants). Indeed, in the present sample, 10 participants consistently chose only high risk or only low risk gambles, which made it impossible to compare their high versus low risk trials dependent on choice. We presented two options on each side also in the no-choice trials in order to control for visual and motor factors, i.e., in both types of trials, choice and no-choice, there were two alternatives and the participants needed to select the right or left alternative. In total this resulted in 18–56 high risk trials depending on choice behavior (i.e., participants with only low risk choices would have 18 no-choice high risk trials), and 18–56 low risk trials. We used the presented level of risk as main independent variable of interest to investigate risk processing in OCD patients versus HC. The percentage of risky choices served as a proxy for risk attitude.

#### Acquisition of Images and Pre-processing

Magnetic Resonance Imaging data were obtained using a 3.0 T Intera MRI scanner (Phillips Healthcare, Best, Netherlands) equipped with a SENSE eight-channel receiver head coil. A spin echo-planar (EPI) sequence sensitive to blood oxygenation level-dependent (BOLD) contrast (TR/TE = 2300/25 ms, matrix size 96 × 96, voxel size 2.29 mm × 2.29 mm × 3 mm, 40 slices, no gap) was used to acquire approximately 254 volumes and a high resolution structural scan was used for anatomical reference with EPI data.

Imaging data were analyzed using Statistical Parametric Mapping (SPM8; Wellcome Trust Centre for Neuroimaging, London, UK). Functional images of each subject were corrected for differences in slice timing, realigned, co-registered with the structural scan, segmented for normalization to an MNI template and resampled at 2 mm× 2 mm × 2 mm. Finally images were smoothed using an 8 mm full width at half maximum Gaussian kernel.

### Data Analysis

#### Behavior

Demographical data and behavioral performance inside and outside the scanner were analyzed using SPSS 19. Group differences in IF and age were analyzed using independent sample *t*-tests, and gender proportions were analyzed using a *χ*^2^ test. The significance level was set at *p* < 0.05. After confirming with the Shapiro–Wilk test that the distributions of risk-aversion and CE (average CE of three risky gambles) in both groups did not significantly deviate from normality, between-group differences were analyzed with an independent sample *t*-test.

RTs in the scanning task were analyzed with a mixed model ANOVA using risk level of trial (high vs. low) as a within-participant variable and group as a between-participant variable. The RTs and percentages of risky choices were compared between groups with an independent sample *t*-test.

#### Neuroimaging

At the first level, a high-pass filter (1/128 Hz) was applied to account for low-frequency signal drift and temporal autocorrelation was modeled as an AR(1) process. The onset of the first period (i.e., presentation of gambles) was modeled with a stick function. The level of risk that participants chose (high or low) was our independent variable of interest and we modeled chosen risk level (high>low) as a parametric modulator for both choice and no-choice trials. Risk level during presentation of gambles was therefore defined by the following behavioral choice. Specifically, we constructed a parametric modulator that assigned a 1 to all chosen high risk trials and a -1 to all chosen low risk trials. Note that in the no-choice trial participants were forced to make either a high or low risk choice by choosing from two identical options. These trials were included to expose participants to both risk levels. Therefore in our variable of interest choice and no-choice trials were used together. The six realignment parameters were included to account for head movement. Subject-specific contrasts were obtained for the parametric modulator and entered into second-level random effects analyses using an independent sample *t*-test to investigate group differences for high > low risk.

In addition at the second-level a linear regression was conducted to determine whether risk attitude (i.e., level of risk-aversion per subject as measured by the behavioral task, CE low – CE high) influences brain activation differently between groups in the high > low risk contrast. Specifically, we used a factorial model ANOVA to assess the risk contrast, with groups (patient/controls) as between-subject factor and risk attitude as subject-specific variable.

Statistical tests were corrected for multiple comparisons across the whole brain at the cluster level (*p* < 0.05, family wise error correction) using a cluster-forming threshold of *p* < 0.01. The figures are presented at a threshold of *p* < 0.005 uncorrected for visualization with the left side of the brain on the right side of the figures.

## Results

### Behavioral Results Outside Scanner

The data showed that the risk attitude measures were similar for both groups: no significant difference between OCD patients and HC in mean risk-aversion or mean risk premium during the risk attitude assessment prior to the scanning session (*p* ≥ 0.25; **Table 2**). In agreement with this, the ranges of risk attitudes were similar in both groups: the highest level of risk-seeking was -15 in patients and -18 in controls while the highest level of risk-aversion was 20 for patients and 24 for controls. These results indicate that OCD patients are not more risk-averse than HC and that both groups are heterogeneous and vary considerably in their risk attitudes.

**Table 2:** Risk attitude and behavioral results of risk processing paradigm.

Risk attitude (outside scanner)	Patients (*N* = 17)		Controls (*N* = 13)		Difference
	Mean *(SD)*	Range	Mean *(SD)*	Range	*P*-value
Risk aversion (high-low premium)	-0.59 (9.4)	-15 to 20	3.46 (12.6)	-18 to 24	0.32

**Risk processing paradigm (inside scanner)**	**Patients (*N* = 18)**		**Controls (*N* = 16)**		**Difference**
	**Mean *(SD)***	**Range**	**Mean *(SD)***	**Range**	***P*-value**

Risky choices (%)	35.73 (32.45)	0–100	22.36 (28.89)	0–100	0.21
RT Risk ^∗^ Group					0.68
RT Risk					0.04^1^
Group: RT high risk (s)	1.625 (0.23)	1.26–2.09	1.54 (0.24)	1.16–1.96	0.29
Group: RT low risk (s)	1.690 (0.29)	1.33–2.41	1.58 (0.30)	1.11–2.15	0.29

*RT, reaction time; ^1^significant (p< 0.05).*

### Behavioral Results Inside Scanner

In line with similar risk attitudes in both groups prior to scanning, we also observed no significant group differences in the percentage of high risk choices during the task in the scanner (**Table 2**). Additionally we found a positive correlation at trend significance (*r* = 0.292 *p* = 0.124) for risk aversion (measured outside scanner) and percentage of safe choices made in choice trials (measured inside scanner).

RTs differed between high risk versus low risk gambles with shorter RTs for selecting the high risk gamble. This indicates that participants respond differently to high risk compared to low risk validating the risk manipulation. However, there were no significant group differences or group × condition interaction effects.

### Neural Correlates of Risk Processing

We examined neural processing of risk while participants were exposed to high versus low risk (**Figure 1**). No significant main effects and no significant differences between patients and controls were found when we compared brain activation induced by high versus low risk. Thus, we found no indications for neural differences in risk processing between the groups.

### Effect of Risk Attitude on Risk Processing

**FIGURE 2:**
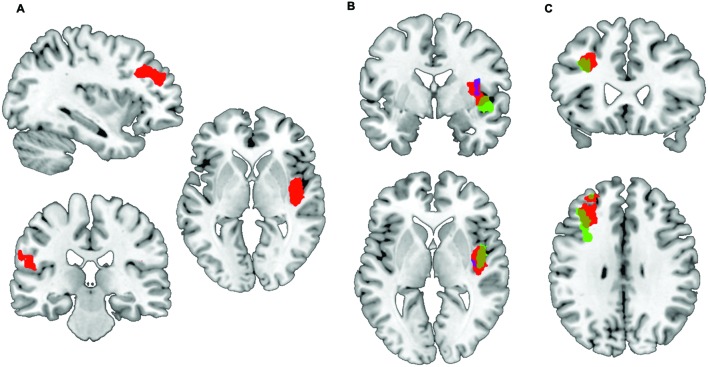
**Preferential correlations between risk-related activation and risk-aversion of patients compared to controls. (A)** Stronger correlation between risk-aversion and brain activation in high risk > low risk contrast in OCD patients compared to healthy participants in the insula, dorsolateral prefrontal cortex, precentral and postcentral gyrus. **(B)** Patients show a positive correlation between insula activation in the high risk > low risk contrast and risk-aversion (green) while controls show a negative correlation (blue); the red cluster reveals the comparison between groups. **(C)** Patients show a positive correlation between dorsolateral prefrontal cortex activation in the high risk > low risk contrast and risk-aversion (green), the red cluster reveals the comparison between patients and controls.

**FIGURE 3:**
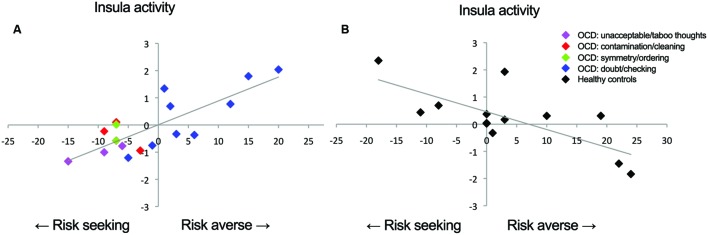
**Correlations between insula activation and risk-aversion.** Positive correlation for **(A)** patients and negative correlation for **(B)** controls between insula activation and risk-aversion. Blue points in **(A)** represent patients within doubt/checking symptom dimension.

To test whether risk attitude affected neural processing of risk differently across groups we performed a linear regression analysis between risk attitude (i.e., level of risk-aversion) and the high versus low risk fMRI contrast. This revealed an interaction effect with group: patients showed a stronger correlation between risk-aversion and brain activation (high risk > low risk) than controls in the insula (*T* = 5.95, *P*_FWE_ < 0.001), dorsolateral prefrontal cortex (*T* = 5.99, *P*_FWE_ < 0.001) and pre- and postcentral gyrus (*T* = 4.33, *P*_FWE_ = 0.001; *T* = 4.23, *P*_FWE_ < 0.032: **Figures 2A** and **3**; **Table 3**; all statistical tests were whole-brain cluster-level corrected). These results remained significant after controlling for differences in anxiety and depression scores. No significantly stronger correlations were found for the controls compared to patients.

**Table 3:** Comparison of regression analysis between groups.

Test	Direction of correlation	Region	Side	Cluster level P value (FWE)	*T*	Cluster size	MNI		
							*X*	*Y*	*Z*
Group comparison	Patients >controls	Insula	R	0.000	5.95	1572	48	-14	12
		DLPFC	L	0.000	5.99	840	-32	28	32
		Precentral gyrus	L	0.001	4.33	682	-64	-30	26
		Pre/postcentral gyrus	L	0.032	4.23	407	-48	-4	44
Controls	Negative	Insula	R	0.047	5.64	304	36	6	12
Patients	Positive	Insula	R	0.001	6.11	758	48	2	-6
		DLPFC	L	0.004	5.57	579	-34	26	30
		Precentral gyrus	L	0.021	4.34	433	-64	-28	30

*Comparison of regression analysis of brain activation in the high-risk > low-risk contrast against risk aversion between groups. R,right; L, left; DLPFC, dorsolateral prefrontal cortex. For overlap between different insula and DLPFC clusters see **Figures 2B,C**.*

In order to determine the direction of the association within groups we performed follow-up testing which showed a positive correlation between insula activity during risk processing and risk-aversion in patients (*T* = 6.11, *P*_FWE_ = 0.001: **Figures 2B** and **3A**; **Table 3**), and a negative correlation between risk-aversion and activation of this region for controls (*T* = 5.64, *P*_FWE_ = 0.047: **Figures 2B** and **3B**; **Table 3**). Patients also showed a positive correlation between risk-aversion and activity in the DLPFC (*T* = 5.57, *P*_FWE_ = 0.004) and activity in the precentral gyrus (*T* = 4.34, *P*_FWE_ = 0.021: **Table 3**; **Figure 2C**).

These results imply that patients and controls show an opposite pattern of insula recruitment during risk processing: high risk situations resulted in higher insula activation in risk-averse patients while high risk situations resulted in lower insula activation in risk-averse HC. Moreover compared to HC, patients showed stronger activation increases in the DLPFC and pre/postcentral gyrus with risk-aversion during risk processing (**Table 3** and **Figures 2A,C**).

### *Post Hoc* Exploration of Risk Attitude and Clinical Data in the Patient Group

The lack of differences in risk attitude between groups may be due to the heterogeneity of the disorder where different subgroups of OCD show different levels of risk attitude. To explore this hypothesis, patients were allocated to one of five OCD symptom dimensions: (i) hoarding, (ii) contamination/cleaning, (iii) symmetry/ordering, (iv) unacceptable/taboo thoughts (v) doubt/checking according to the YBOCS symptoms checklist (Brakoulias et al., 2013). When patients scored on multiple dimensions, they were assigned to the dimension for which they reported most symptoms. We excluded patients with predominantly hoarding symptoms, as there is evidence that this dimension may be independent from OCD (Pertusa et al., 2008). All seven risk-averse OCD patients belonged to the doubt/checking subgroup, whereas the 10 patients in the risk-seeking group consisted of three patients with mainly unacceptable/taboo thoughts, two with mainly symmetry/ordering symptoms, three with mainly contamination/cleaning symptoms, and only two with mainly doubt/checking symptoms (Supplementary Table S1). Note that the two risk-seeking patients with mainly doubt/checking symptoms were close to being risk-neutral. Accordingly, risk-averse and risk-seeking patients showed a significant difference in symptom dimension (*p* = 0.014). On average, patients with doubt/checking symptoms were significantly more risk-averse than patients with other symptoms (*p* < 0.001), and this remained significant after controlling for whether patients used medication (*p* = 0.001), indicating that the differences between groups were not due to differences in medication use.

To test whether medication use affected risk attitude or the number of risky choices in the scanner we used an independent sample *t*-test to compare medicated and unmedicated OCD patients. We found no differences in mean risk-aversion or percentage of risky choices in the scanner between medicated and unmedicated OCD patients: -1.3 (10.4) vs. 0.7 (7.9) (*p* = 0.70); risky choices, medicated and unmedicated patients: 39.4% (37.9) vs. 28.3% (18.1) (*p* = 0.41).

## Discussion

Contrary to common belief, patients with OCD were not more averse to risk than HC in the present task. Regardless, HC and OCD patients showed an opposite correlation between risk-aversion and insula activity during risk processing: insula activity correlated positively with risk-aversion in patients, whereas in HC insula activity correlated negatively with risk-aversion. Moreover OCD patients showed stronger activation increases in the DLPFC and pre/postcentral gyrus with risk-aversion during risk processing.

Patients showed stronger activation in the right insula to high versus low risk with increasing risk-aversion, whereas controls showed stronger activation in the same region with increasing propensity to seek risk (i.e., decreasing risk-aversion). Growing evidence suggests that the insula is involved in interoceptive processing (i.e., perception of internal feelings of the body) and the evaluation of interoceptive states contributing to subjective feelings and emotions (Craig, 2002). Additionally neuroeconomic studies have pointed to a role of the insula in risk processing (Kuhnen and Knutson, 2005; Huettel, 2006; Preuschoff et al., 2008; Burke and Tobler, 2011). Risk processing in humans is not only a deliberate calculation of probability but also involves the evaluation of affective states (Mukherjee, 2010). Taken together these findings have led to the hypothesis that the insula is critically involved in the affective processes underlying risk processing (Paulus et al., 2003; Gowin et al., 2014). Additionally the insula has been related to cognitive control and the accumulation of information in the process of decision making possibly affecting risk taking (Hu et al., 2014). Our data concur and raise the possibility that interoceptive processes may play a particularly prominent role in the subgroup of OCD patients with doubt and checking symptoms.

Moreover, the insula may also be involved in expressing the affective components of risk into behavior. In agreement with this notion, evidence suggests that activity in the insula may be associated with an urge for risk taking in HC (Xue et al., 2010) and in non-human species (Ishii et al., 2012) which is in line with our finding that risk-related insula activation is associated with risk-seeking in HC. The finding that activity in the same region of the insula in patients is correlated with risk-aversion may suggest that at least some parts of the insula assume a differential role in the two groups: for HC this subregion may bias behavior towards taking risks whereas for OCD patients it could bias behavior towards risk avoidance. Alternatively, in both groups the insula may signal general arousal or decision urgency but this is experienced more strongly by risk-averse patients and by risk-seeking HC participants. In both cases, the insula seems to play an important role in the integration of bodily interoceptive signals with awareness appropriate action tendencies (i.e., approach or avoid) in the face of high risk. The insula may play such a role (Xue et al., 2010; Ishii et al., 2012) in an individually adjusted manner (Paulus et al., 2003; Kuhnen and Knutson, 2005).

Additionally, risk-averse OCD patients showed increased recruitment of the DLPFC and precentral gyrus during risk processing in contrast to HCs. This contrasts with HCs (Tobler et al., 2009; Holper et al., 2014) and may reflect increased collaboration between prefrontal regions and the insula when patients process risk. The DLPFC has previously been shown to encode the value of risk (Huettel, 2006; Christopoulos et al., 2009; Tobler et al., 2009; Holper et al., 2014). Moreover DLPFC has been implicated in executive functioning and specifically cognitive control (Mohr et al., 2010). Speculatively, the increased recruitment of this region in risk-averse OCD patients might show additional control mechanisms in the face of risky choices.

No differences in risk attitude were found between groups, suggesting that in general, OCD patients are not more risk averse than HCs. Consistent with the behavioral results no overall neural differences were found during risk processing between groups. A possible explanation for this lack of differences between groups but the finding that the groups differ in correlation between risk attitude and neural correlates is that OCD is a heterogeneous disorder and different subtypes of OCD show differences in risk attitude. Indeed *post hoc* analyses showed that all risk-averse OCD patients expressed doubt/checking symptoms and this subgroup was more risk-averse than patients with other symptoms. Patients with doubt/checking symptoms report obsessions about causing unintentional harm to others, fear that something terrible might happen, indecisiveness and checking compulsions. Interestingly, the heightened risk-aversion of this group became apparent here even in situations in which only gains could occur. The finding that risk-aversion may contribute to only a specific subtype of OCD suggests that for this group addressing abnormal risk-assessment in cognitive behavioral therapy may be helpful.

Several limitations are worth mentioning. One potential limitation of our study was the relatively strong behavioral consistency of our subjects, which made it impossible to investigate potential interactions between risk and choice. Additionally, in the paradigm used in this study, risk arose from the variance of money that could be earned while there was no risk of losing money. Therefore risk-aversion was not based on loss prevention but on a preference for more certainty in gain. In a pure gain context, risk-aversion could result from perceiving the lowest possible outcome as relative loss or from perceiving more variance. OCD patients may have different neural responses during actual loss versus reward anticipation (Choi et al., 2012) and including losses could have affected risky choices in OCD irrespective of symptoms. However, a previous study (Gillan et al., 2013) using both gains and losses nevertheless confirmed our result that on average risk processing is unaffected in OCD. A further limitation could be that the range between risk-seeking and risk-averse extremes was higher in both the OCD and HC group than expected based on a previous study using a similar task (Christopoulos et al., 2009). This increased variance in risk attitude may be due to the heterogeneity of our group in terms of age and IF compared to the group of primarily college students used in the previous study.

Another potential limitation is the fact that 10 patients were using serotonin-reuptake inhibitors (SRIs) and one patient was using a tricyclic antidepressant whereas 7 other patients did not receive medication. Serotonin neurotransmission is correlated with successful withholding of responses and risk avoidance, whereas low serotonin promotes early responding and risk taking (Cools et al., 2008; Long et al., 2009). In the present study we did not find any differences in risk-aversion or propensity for risky choice between medicated and unmedicated patients and differences in risk-aversion between patients with doubt/checking symptoms and other patients remained significant after controlling for medication use. Therefore it seems unlikely that in our study SRI medication explains the risk profiles of patients. However, for comparisons within the OCD group the sample size is small and this has to be taken into account when interpreting the differences between risk-averse and risk-seeking patients.

In conclusion, we found elevated insula activation during risk processing in risk-averse OCD patients, which may suggest that the insula is involved in an increased urge to avoid risk in these patients. Increased avoidance signaling in the insula might contribute to the development of risk-avoidant strategies in this group, which in turn could lead to persistence of the disorder.

## Author Contributions

JL analyzed the data and wrote the manuscript. MF initiated the study and revised the manuscript. PT designed the experiment and revised the paper. GW drafted and revised the paper. WB revised the paper. BK collected the data. DD initiated the study and revised the paper.

## Conflict of Interest Statement

The authors declare that the research was conducted in the absence of any commercial or financial relationships that could be construed as a potential conflict of interest.
