# Supplementary material for: Doubt in the Insula: Risk Processing in Obsessive-Compulsive Disorder
Source: Front Hum Neurosci. 2016 Jun 14;10:283. doi: 10.3389/fnhum.2016.00283 (PMC4905944; doi:10.3389/fnhum.2016.00283)
Supplement: Supplementary file 1 [file Table_1.DOCX]

| **Pt nr** |  | **Gender** | **Age** | **Handedness** | **Y-BOCS** | **HAMA** | **HAMD** | **Age onset** | **Duration (y)** | **Medication** | **Risk aversion** | **Symptom dimension** |
| --- | --- | --- | --- | --- | --- | --- | --- | --- | --- | --- | --- | --- |
| 1 | 201 | M | 34 | r | 17 | 13 | 12 | 16 | 18 | SSRI | -15 | Unacceptable/taboo thoughts |
| 2 | 202 | M | 54 | r | 26 | 17 | 10 | 23 | 31 | SSRI | -9 | Contamination/cleaning |
| 3 | 205 | F | 28 | r | 33 | 24 | 24 | 8 | 20 | SSRI | -9 | Unacceptable/taboo thoughts |
| 4 | 218 | F | 30 | r | 21 | 12 | 2 | 18 | 12 | none | -7 | Symmetry /ordering |
| 5 | 204 | F | 34 | r | 30 | 15 | 16 | ? |  | SSRI | -7 | Contamination/cleaning |
| 6 | 222 | F | 41 | r | 30 | 9 | 9 | 29 | 5 | SSRI | -7 | Symmetry /ordering |
| 7 | 203 | F | 23 | r | 20 | 15 | 6 | 18 | 5 | none | -6 | Contamination/cleaning |
| 8 | 221 | M | 41 | r | 29 | 1 | 2 | 15 | 26 | SSRI | -5 | Doubt/checking |
| 9 | 211 | M | 32 | r | 12 | 8 | 5 | 17 | 15 | SSRI | -3 | Unacceptable/taboo thoughts |
| 10 | 223 | F | 36 | l | 26 | 4 | 6 | 20 | 16 | None | -1 | Doubt/checking |
| 11 | 213 | M | 37 | l | 16 | 8 | 4 | 15 | 22 | None | 1 | Doubt/checking |
| 12 | 214 | F | 29 | l | 18 | 1 | 2 | 5 | 24 | None | 2 | Doubt/checking |
| 13 | 219 | M | 31 | r | 32 | 16 | 15 | 12 | 17 | None | 3 | Doubt/checking |
| 14 | 209 | F | 28 | r | 24 | 26 | 22 | 10 | 18 | SSRI | 6 | Doubt/checking |
| 15 | 212 | F | 36 | r | 19 | 15 | 11 | 25 | 11 | SSRI | 12 | Doubt/checking |
| 16 | 217 | F | 34 | r | 20 | 0 | 2 | 12 | 22 | None | 15 | Doubt/checking |
| 17 | 208 | F | 30 | r | 26 | 17 | 15 | 20 | 10 | SSRI | 20 | Doubt/checking |
| 18 | 215 | F | 34 | r | 31 | 0 | 0 | 32 | 2 | TCA | N/A | Symmetry /ordering |

**Table S1**

Clinical data OCD patients

HAM-A: Hamilton Ratings Scale for Anxiety; HAMD: Hamilton Ratings Scale for Depression; YBOCS: Yale-Brown Obsessive-Compulsive Scale; M: male; F: Female; SSRI: selective serotonin reuptake inhibitor; TCA: tricyclic antidepressants; N/A: not available
